# Isolated acute pseudobulbar palsy with infarction of artery of percheron: case report and literature review

**DOI:** 10.4314/ahs.v21i1.22

**Published:** 2021-03

**Authors:** Jamir Pitton Rissardo, Ana Fornari Caprara

**Affiliations:** Federal University of Santa Maria, Santa Maria, Rio Grande do Sul, Brazil

**Keywords:** Pseudobulbar palsy, thalamus, infarction

## Abstract

**Introduction:**

Pseudobulbar palsy (PBP) is characterized by supranuclear lesions in the corticobulbar pathway. Neoplasia, inflammatory, demyelinating, and stroke are possible etiologies of this disorder.

**Case report:**

We report an elderly female who presented with dysarthria. She was dysarthric with a hypernasal voice, no apraxia or aphasia was observed. Tongue movements were slow with limited amplitude. Her soft palate dropped bilaterally; gag reflex was present. Also, she reported swallowing difficulty and choking with her saliva. Bilateral vertical and horizontal gaze were intact to either voluntary or oculocephalic movements. A cranial CT scan was suggestive of artery of Percheron (AOP) infarction. Brain magnetic resonance imaging showed hypersignal on diffusion-weighted and T2-weighted images and hyposignal on apparent diffusion coefficient in both thalami. CT angiography scan revealed an AOP originating from the left posterior cerebral artery. The swallowing study with a videofluoroscopic demonstrated oral and pharyngeal phases with severe dysfunction.

**Conclusion:**

To the authors' knowledge, there are two cases of individuals with artery of Percheron infarction who developed PBP associated with other clinical syndromes. Still, isolated PBP following infarction of Percheron's artery was not reported. We hypothesized that the PBP may have occurred because of the existence of vascular territory variations in the perforating arteries that arise from the AOP.

## Introduction

Pseudobulbar palsy (PBP) is characterized by supranuclear lesions in the corticobulbar pathway. Neoplasia, inflammatory, and demyelinating diseases are possible etiologies of this disorder. In this context, another common cause of PBP are multiple and recurrent strokes.^1^ But, infarction of the diencephalon leading to dysarthria and dysphonia is rare.

There are only a few cases of bilateral paramedian thalamic infarction associated with acute pseudobulbar symptoms that have been reported in the literature. More specifically, to the authors' knowledge, there are two case reports of individuals with artery of Percheron (AOP) stroke who developed PBP associated with other clinical syndromes.^2^,^3^ Still, isolated PBP following infarction of Percheron's artery was not reported.

Herein, we report a case of an adult female who developed isolated PBP secondary to infarction of AOP.

## Case report

A 70-year-old female presenting with a difficult and unclear articulation of speech with sudden onset was admitted to our hospital. The individual was previously healthy, retired, and her family history was negative for neurological diseases. Her vital signs (blood pressure, heart rate, respiratory rate, temperature) were normal and stable.

On neurological examination, she was dysarthric with a hypernasal voice, no apraxia or aphasia was observed. Tongue movements were slow with limited amplitude and because of this it seemed that the tongue had difficulty moving, but when it was protruded no deviation occurred. Her soft palate dropped bilaterally; gag reflex was present. Also, she reported swallowing difficulty and choking with her saliva. Bilateral vertical and horizontal gaze were intact to either voluntary or oculocephalic movements. Palpebral oculogyric reflex was normal. Myosis, ptosis, nystagmus, and skew deviation were not present. On the National Institutes of Health Stroke Scale (NIHSS), she only scored in dysarthria, which was characterized by unintelligible slurring or out of proportion to dysphasia.

Laboratory blood tests and urinalysis were within normal limits. A cranial computed tomography (CT) scan at the symptoms' onset (Figure 1 – A, D) and two days after (Figure 1 – B, C, E, F) was suggestive of infarction of Percheron's artery. Brain magnetic resonance imaging showed hypersignal on diffusion-weighted and T2-weighted images and hyposignal on apparent diffusion coefficient in both thalami regions. CT angiography scan revealed an artery of Percheron originating from the left posterior cerebral artery, which was impaired. The cerebral spinal fluid analysis was normal; culture was negative. The swallowing study with a videofluoroscopic demonstrated oral and pharyngeal phases with severe dysfunction. The patient was referred to another hospital and the follow-up was lost.

**Figure:**
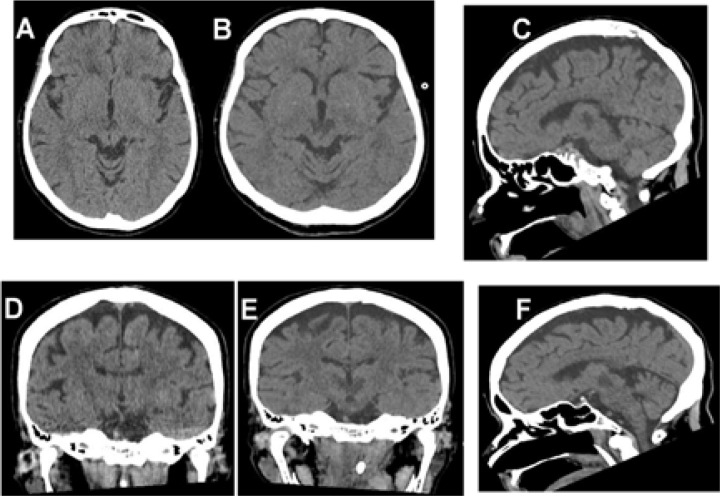


## Discussion

The thalamus is a complex structure formed by nuclei that integrate sensory, motor, and behavioral signals of the cerebral cortex with other pathways. Although there are significant variations and overlaps, this diencephalic large gray matter is supplied by four arteries: three derived from the vertebrobasilar system (paramedian thalamo-subthalamic, thalamogeniculate, and posterior choroidal arteries) and one derived from the posterior communicating artery (polar artery). The paramedian arteries are the most variable when we evaluate number, size, and territorial contribution to the thalamus. 4,5

Percheron described three possible variations involving the paramedian thalamic-mesencephalic arterial supply. The most common variation is small perforating arteries arising from the first segment of the posterior cerebral artery (PCA). The second variant consists in the dependency of an isolated terminal branch nascent in the PCA for the bilateral blood supply of paramedian thalamus and rostral midbrain. Third, perforating arteries arising from a branch bridging the first segments of both PCA.^6^

The second type, also known as the artery of Percheron (AOP), is considered a normal variant. The prevalence varies among different studies ranging from five to thirty percent in different populations. The classic clinical triad of symptoms in the infarction of the AOP is characterized by consciousness impairment, vertical gaze palsy, and cognitive or behavioral disturbances but a great variability of presentations was already described.^7^

Only a few cases of patients with bilateral paramedian thalamic infarction who develop acute pseudobulbar palsy have been reported in the literature. We identified two cases after a review of the English-language published literature and we compared them with the present case (Table 1).^2^,^3^,^8^ To the authors' knowledge, the reported cases presented other clinical syndromes associated with the pseudobulbar symptoms. In this way, the present case is the first to report isolated pseudobulbar palsy following infarction of Percheron's artery. A literature search was performed in Embase, Google Scholar, Lilacs, Medline, Scielo, and Science Direct, using a set of terms that included the thalamus, thalamic stroke, stroke, and abducens palsy.

**Table 1:** Case Reports of Patients with Bilateral Paramedian Thalamic Stroke Who Developed Acute Pseudobulbar Palsy

References		Karacostas et al	Lee et al	Present
Country		1994	2016	2019
Year		Greece	Korea	Brazil
Age (y)/Sex		28/F	61/M	70/F
Initial symptoms		Dysarthria, dysphagia, hypernasal voice and mild right arm paresis	Sudden-onset dysarthria and dysphagia without motor damage	Difficult articulation of speech
Comorbidities and medications in use		Contraceptives and smoking	Alcoholic, hypertension, diabetes mellitus	None
Acute pseudobulbar palsy characteristics	Neurological exam	-	Mild cognition impairment, upward gaze limitation, dropped soft palate, dysarthric, hypernas al voice, slow tongue movements, dysmetria in upper limbs	Dysarthria, hypernasal voice, dropped soft palate, slow tongue movements
	Thurel classification^8^	Pontine	Cortical with ataxia and cognitive impairment probably due to alcoholism	Cortical
	Facio, pharyngo, and glossomasticatory Diplegia	Yes	Yes	Yes
	Automatic voluntary dissociation	Yes	Yes	Yes
	Pyramidal signs	Yes	No	No
	Emotional lability	NR	NR	No
	Intellectual impairment	NR	Yes	No
	Cerebellar signs	NR	Yes	No
Neuroimaging		MRI	CT, MRI, CTA	CT, MRI, CTA
CT, MRI findings		Bilateral isolated thalamic infarcts	Lesion limited to the thalamus	Bilateral thalamic with extension to midbrain
CTA findings		-	Artery of Percheron	Artery of Percheron
Follow-up		-	31 days, patient tolerate dysphagia diets 2&3, moderate dysarthria, 50% intelligible speech	Patient was referred. The follow-up was lost.

Abbreviations: CT, computed tomography scan; CTA, computed tomography angiography scan F, female; M, Male; MRI, magnetic resonance imaging; NR, not reported; y, year-old.

In the cases of Table 1, all the subjects had acute pseudobulbar palsy but two of them also presented other clinical syndromes at the same time. The study of Karacostas et al presented an individual with mild right arm paresis, which is commonly associated with PBP due to extend lesions affecting the upper motor neuron pathway.^3^ In the second study, the subject had cerebellar signs and bilateral upward gaze palsy, which suggests that the infarction also compromised the midbrain; although the authors reported that the mesencephalon was not damaged providing an axial image in fluid-attenuated inversion recovery.^2^ However, only coronal and mainly sagittal neuroimaging are able to clearly visualize the midbrain structures.^9^

Any lesion in the corticobulbar tract can lead to the clinical manifestation of PBP characterized by dysarthria, dysphagia and nasal tuning speech. In this way, we hypothesized that the PBP may have occurred in the subjects of Table 1 because of the existence of vascular territory variations in the perforating arteries that arise from the AOP (Figure 2).^4^,^5^,^7^ The posterior limb of internal capsule contains part of the corticospinal, cerebellar and pontine pathways.^10^,^11^ In fact, diffusion tensor imaging and voltage stimulation variation studies suggested that fronto-ponto-cerebellar fibers are located medially to the corticospinal fibers.^12^,^13^ In addition, some patients with AOP may have perforating branches throughout their passage from the midbrain to the thalamus. Thus, pseudobulbar, pyramidal, and cerebellar signs could occur in a Percheron's artery infarction. Moreover, the corticobulbar tract is probably the most common to be affected, which could explain the occurrence of the isolated PBP in only one report.^7^

**Figure:**
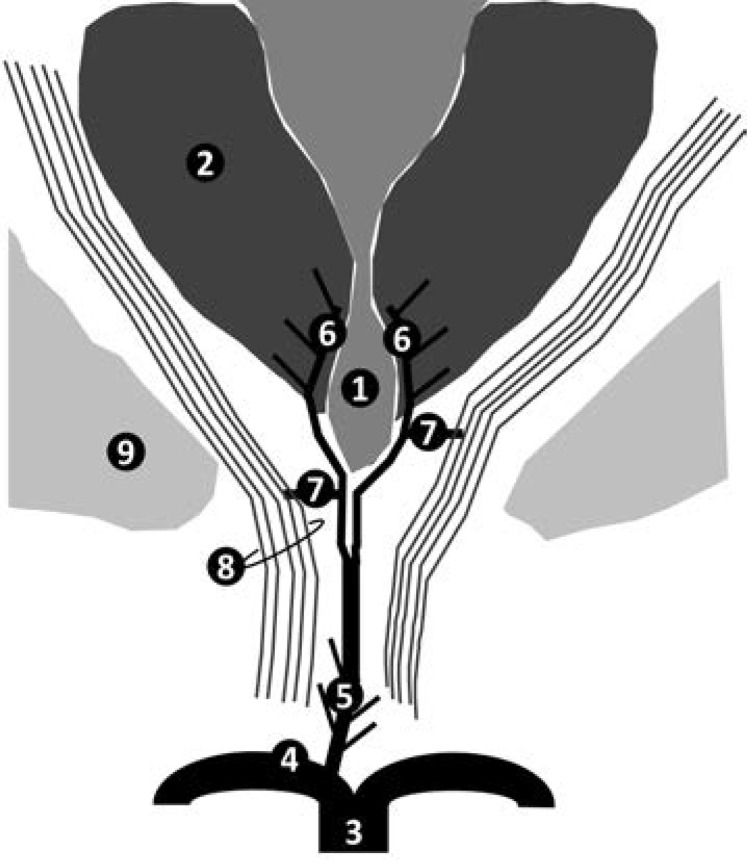


Karacostas et al and Lee et al proposed a different pathophysiological mechanism to this presentation.^2^,^3^ The ventrolateral nucleus of thalamus has connections with^4^, ^6^, and face cortical areas. Therefore, they proposed that the bilateral lesions in these nuclei could interrupt the thalamocortical projections to the face, resulting in acute pseudobulbar palsy via diaschisis. 14 Furthermore, Lee et al hypothesized that the oculomotor symptoms occurred because of a lesion affecting the anteroventral nuclei; however, this structure is supplied by the tuberothalamic branch that arises from the middle third of the posterior communicating artery so only large thalamic damage would lead to all these clinical manifestations.^4^
